# Diiodido{2-(morpholin-4-yl)-*N*-[1-(2-pyrid­yl)ethyl­idene]ethanamine-κ^3^ *N*,*N*′,*N*′′}zinc

**DOI:** 10.1107/S1600536811014656

**Published:** 2011-04-22

**Authors:** Nura Suleiman Gwaram, Hamid Khaledi, Hapipah Mohd Ali

**Affiliations:** a Department of Chemistry, University of Malaya, 50603 Kuala Lumpur, Malaysia

## Abstract

In the title compound, [ZnI_2_(C_13_H_19_N_3_O)], the Zn^II^ ion is five-coordinated in a distorted square-pyramidal geometry, in which the basal plane is defined by three N atoms from the Schiff base ligand and one iodide ion. A second iodide ligand, situated in the apical position, completes the coordination geometry. In the crystal structure, C—H⋯O hydrogen bonds link a pair of mol­ecules around an inversion centre into a dimer.

## Related literature

For the structure of an analogous ZnCl_2_ complex, see: Ikmal Hisham *et al.* (2011 ▶). For square-pyramidal ZnI_2_ complexes with *N*,*N*′,*N*′′-tridentate ligands, see: Drew & Hollis (1978 ▶); Yousefi (2010 ▶). For a description of the geometry of complexes with five-coordinated metal ions, see: Addison *et al.* (1984 ▶).
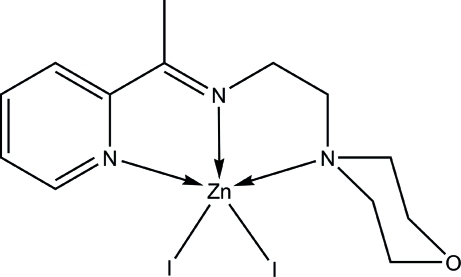


## Experimental

### 

#### Crystal data

[ZnI_2_(C_13_H_19_N_3_O)]
*M* *_r_* = 552.48
Triclinic, 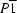

*a* = 8.8874 (3) Å
*b* = 10.3117 (4) Å
*c* = 10.3643 (4) Å
α = 68.8810 (18)°
β = 81.959 (2)°
γ = 66.3990 (17)°
*V* = 811.91 (6) Å^3^
*Z* = 2
Mo *K*α radiation
μ = 5.31 mm^−1^
*T* = 100 K
0.17 × 0.13 × 0.09 mm

#### Data collection

Bruker APEXII CCD diffractometer
Absorption correction: multi-scan (*SADABS*; Sheldrick, 1996 ▶) *T*_min_ = 0.465, *T*_max_ = 0.646
7292 measured reflections
3517 independent reflections
3260 reflections with *I* > 2σ(*I*)
*R*_int_ = 0.013

#### Refinement

*R*[*F* ^2^ > 2σ(*F* ^2^)] = 0.018
*wR*(*F* ^2^) = 0.045
*S* = 1.05
3517 reflections
182 parameters
H-atom parameters constrained
Δρ_max_ = 0.85 e Å^−3^
Δρ_min_ = −1.23 e Å^−3^

### 

Data collection: *APEX2* (Bruker, 2007 ▶); cell refinement: *SAINT* (Bruker, 2007 ▶); data reduction: *SAINT*; program(s) used to solve structure: *SHELXS97* (Sheldrick, 2008 ▶); program(s) used to refine structure: *SHELXL97* (Sheldrick, 2008 ▶); molecular graphics: *X-SEED* (Barbour, 2001 ▶); software used to prepare material for publication: *SHELXL97* and *publCIF* (Westrip, 2010 ▶).

## Supplementary Material

Crystal structure: contains datablocks I, global. DOI: 10.1107/S1600536811014656/hy2422sup1.cif

Structure factors: contains datablocks I. DOI: 10.1107/S1600536811014656/hy2422Isup2.hkl

Additional supplementary materials:  crystallographic information; 3D view; checkCIF report

## Figures and Tables

**Table 1:** Selected bond lengths (Å)

Zn1—N1	2.205 (2)
Zn1—N2	2.093 (2)
Zn1—N3	2.269 (2)
Zn1—I1	2.6018 (4)
Zn1—I2	2.6506 (4)

**Table 2:** Hydrogen-bond geometry (Å, °)

*D*—H⋯*A*	*D*—H	H⋯*A*	*D*⋯*A*	*D*—H⋯*A*
C13—H13*A*⋯O1^i^	0.99	2.55	3.491 (3)	159

Symmetry code: (i) 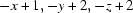.

**Fig. 1.**
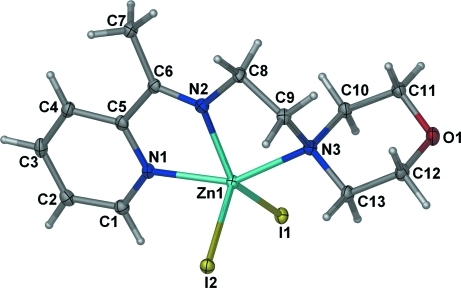
Molecular structure of the title compound. Displacement ellipsoids are drawn at the 50% probability level.

## supplementary crystallographic information

### Comment

The title compound (Fig. 1) was obtained *via* the complexation of Zn^II^ ion with the *in situ* prepared Schiff base, 2-morpholino-*N*-[1-(2-pyridyl)ethylidene]ethanamine, and two iodide ions. Similar to what was observed in an analogous ZnCl_2_ complex (Ikmal Hisham *et al.*, 2011), the Schiff base acts as an *N*,*N*',*N*''-tridentate chelate ligand, along with two halide ligands, make a distorted square-pyramidal geometry around the metal ion (τ = 0.24, Addison *et al.*, 1984). The Zn—I and Zn—N interatomic distances (Table 1) are comparable to the values reported for similar structures (Drew & Hollis, 1978; Yousefi, 2010). In the crystal, a pair of the molecules, related by a symmetry operation -*x* + 1, -*y* + 2, -*z* + 2, are linked through C—H···O hydrogen bonds into a centrosymmetric dimer (Table 2).

### Experimental

A mixture of 2-acetylpyridine (0.20 g, 1.65 mmol) and 4-(2-aminoethyl)morpholine (0.21 g, 1.65 mmol) in ethanol (20 ml) was refluxed for 2 h, followed by addition of a solution of zinc(II) acetate dihydrate (0.36 g, 1.65 mmol) and potassium iodide (0.54 g, 3.3 mmol) in a minimum amount of water. The resulting solution was refluxed for 30 min and then left at room temperature. Brown crystals of the title complex were obtained in a few days.

### Refinement

H atoms were placed at calculated positions and refined as riding atoms, with C—H = 0.95 (aryl), 0.98 (methyl) and 0.99 (methylene) Å and with *U*_iso_(H) = 1.2(1.5 for methyl)*U*_eq_(C).

### Crystal data

**Table:**

[ZnI_2_(C_13_H_19_N_3_O)]	*Z* = 2
*M**_r_* = 552.48	*F*(000) = 524
Triclinic, *P*1	*D*_x_ = 2.260 Mg m^−^^3^
Hall symbol: -P 1	Mo *K*α radiation, λ = 0.71073 Å
*a* = 8.8874 (3) Å	Cell parameters from 6030 reflections
*b* = 10.3117 (4) Å	θ = 2.3–30.4°
*c* = 10.3643 (4) Å	µ = 5.31 mm^−^^1^
α = 68.8810 (18)°	*T* = 100 K
β = 81.959 (2)°	Block, brown
γ = 66.3990 (17)°	0.17 × 0.13 × 0.09 mm
*V* = 811.91 (6) Å^3^	

### Data collection

**Table:**

Bruker APEXII CCD diffractometer	3517 independent reflections
Radiation source: fine-focus sealed tube	3260 reflections with *I* > 2σ(*I*)
graphite	*R*_int_ = 0.013
φ and ω scans	θ_max_ = 27.0°, θ_min_ = 2.1°
Absorption correction: multi-scan (*SADABS*; Sheldrick, 1996)	*h* = −11→11
*T*_min_ = 0.465, *T*_max_ = 0.646	*k* = −13→13
7292 measured reflections	*l* = −13→13

### Refinement

**Table:**

Refinement on *F*^2^	Primary atom site location: structure-invariant direct methods
Least-squares matrix: full	Secondary atom site location: difference Fourier map
*R*[*F*^2^ > 2σ(*F*^2^)] = 0.018	Hydrogen site location: inferred from neighbouring sites
*wR*(*F*^2^) = 0.045	H-atom parameters constrained
*S* = 1.05	*w* = 1/[σ^2^(*F*_o_^2^) + (0.0197*P*)^2^ + 1.3278*P*] where *P* = (*F*_o_^2^ + 2*F*_c_^2^)/3
3517 reflections	(Δ/σ)_max_ = 0.002
182 parameters	Δρ_max_ = 0.85 e Å^−^^3^
0 restraints	Δρ_min_ = −1.23 e Å^−^^3^

### Fractional atomic coordinates and isotropic or equivalent isotropic displacement parameters (Å^2^)

**Table:**

	*x*	*y*	*z*	*U*_iso_*/*U*_eq_	
Zn1	0.77476 (4)	0.81170 (3)	0.66161 (3)	0.01267 (7)	
I1	0.64389 (2)	1.103488 (18)	0.584395 (17)	0.01508 (5)	
I2	1.05998 (2)	0.694938 (19)	0.791314 (17)	0.01566 (5)	
O1	0.3717 (2)	0.8562 (2)	1.0506 (2)	0.0212 (4)	
N1	0.8699 (3)	0.8016 (2)	0.4558 (2)	0.0135 (4)	
N2	0.7235 (3)	0.6403 (2)	0.6380 (2)	0.0137 (4)	
N3	0.6122 (3)	0.7619 (2)	0.8469 (2)	0.0137 (4)	
C1	0.9527 (3)	0.8810 (3)	0.3702 (3)	0.0161 (5)	
H1	0.9697	0.9537	0.3959	0.019*	
C2	1.0153 (3)	0.8615 (3)	0.2446 (3)	0.0181 (5)	
H2	1.0767	0.9176	0.1868	0.022*	
C3	0.9859 (3)	0.7587 (3)	0.2062 (3)	0.0190 (6)	
H3	1.0239	0.7455	0.1197	0.023*	
C4	0.9002 (3)	0.6744 (3)	0.2951 (3)	0.0165 (5)	
H4	0.8797	0.6024	0.2709	0.020*	
C5	0.8456 (3)	0.6981 (3)	0.4199 (3)	0.0145 (5)	
C6	0.7574 (3)	0.6108 (3)	0.5253 (3)	0.0139 (5)	
C7	0.7137 (3)	0.5006 (3)	0.4920 (3)	0.0181 (5)	
H7A	0.6732	0.4412	0.5754	0.027*	
H7B	0.8112	0.4336	0.4582	0.027*	
H7C	0.6280	0.5547	0.4204	0.027*	
C8	0.6366 (3)	0.5663 (3)	0.7513 (3)	0.0161 (5)	
H8A	0.6837	0.4566	0.7693	0.019*	
H8B	0.5189	0.6052	0.7275	0.019*	
C9	0.6557 (3)	0.5993 (3)	0.8785 (3)	0.0150 (5)	
H9A	0.5839	0.5643	0.9531	0.018*	
H9B	0.7707	0.5435	0.9119	0.018*	
C10	0.4339 (3)	0.8512 (3)	0.8159 (3)	0.0157 (5)	
H10A	0.4008	0.8254	0.7440	0.019*	
H10B	0.4152	0.9594	0.7782	0.019*	
C11	0.3273 (3)	0.8231 (3)	0.9429 (3)	0.0177 (5)	
H11A	0.2107	0.8864	0.9174	0.021*	
H11B	0.3391	0.7166	0.9766	0.021*	
C12	0.5400 (3)	0.7684 (3)	1.0885 (3)	0.0188 (6)	
H12A	0.5561	0.6608	1.1276	0.023*	
H12B	0.5690	0.7960	1.1610	0.023*	
C13	0.6531 (3)	0.7918 (3)	0.9652 (3)	0.0168 (5)	
H13A	0.6466	0.8967	0.9338	0.020*	
H13B	0.7676	0.7247	0.9950	0.020*	

### Atomic displacement parameters (Å^2^)

**Table:**

	*U*^11^	*U*^22^	*U*^33^	*U*^12^	*U*^13^	*U*^23^
Zn1	0.01377 (14)	0.01207 (14)	0.01168 (14)	−0.00432 (11)	0.00086 (11)	−0.00440 (11)
I1	0.01607 (9)	0.01218 (8)	0.01448 (9)	−0.00349 (6)	0.00078 (6)	−0.00401 (6)
I2	0.01406 (9)	0.01676 (9)	0.01511 (9)	−0.00421 (7)	−0.00121 (6)	−0.00553 (7)
O1	0.0184 (10)	0.0264 (11)	0.0200 (10)	−0.0062 (8)	0.0039 (8)	−0.0130 (9)
N1	0.0131 (10)	0.0134 (10)	0.0120 (10)	−0.0034 (8)	0.0003 (8)	−0.0039 (8)
N2	0.0130 (10)	0.0110 (10)	0.0147 (11)	−0.0028 (8)	−0.0011 (8)	−0.0033 (8)
N3	0.0150 (11)	0.0130 (10)	0.0116 (10)	−0.0047 (9)	0.0001 (8)	−0.0032 (8)
C1	0.0148 (12)	0.0163 (12)	0.0164 (13)	−0.0051 (10)	−0.0007 (10)	−0.0051 (10)
C2	0.0142 (12)	0.0194 (13)	0.0178 (13)	−0.0053 (11)	0.0010 (10)	−0.0047 (11)
C3	0.0171 (13)	0.0214 (14)	0.0138 (13)	−0.0024 (11)	0.0017 (10)	−0.0067 (11)
C4	0.0153 (13)	0.0178 (13)	0.0160 (13)	−0.0034 (10)	−0.0007 (10)	−0.0084 (11)
C5	0.0108 (12)	0.0140 (12)	0.0160 (13)	−0.0014 (10)	−0.0011 (9)	−0.0053 (10)
C6	0.0116 (12)	0.0123 (12)	0.0159 (13)	−0.0012 (10)	−0.0012 (9)	−0.0057 (10)
C7	0.0187 (13)	0.0189 (13)	0.0185 (14)	−0.0070 (11)	0.0010 (10)	−0.0085 (11)
C8	0.0176 (13)	0.0155 (12)	0.0165 (13)	−0.0077 (10)	0.0038 (10)	−0.0064 (10)
C9	0.0167 (13)	0.0123 (12)	0.0135 (13)	−0.0048 (10)	0.0009 (10)	−0.0028 (10)
C10	0.0145 (12)	0.0169 (12)	0.0148 (13)	−0.0042 (10)	0.0003 (10)	−0.0061 (10)
C11	0.0151 (13)	0.0192 (13)	0.0186 (14)	−0.0055 (11)	0.0020 (10)	−0.0079 (11)
C12	0.0183 (13)	0.0256 (14)	0.0123 (13)	−0.0071 (11)	0.0008 (10)	−0.0076 (11)
C13	0.0181 (13)	0.0197 (13)	0.0124 (13)	−0.0064 (11)	0.0018 (10)	−0.0067 (10)

### Geometric parameters (Å, °)

**Table:**

Zn1—N1	2.205 (2)	C4—H4	0.9500
Zn1—N2	2.093 (2)	C5—C6	1.495 (4)
Zn1—N3	2.269 (2)	C6—C7	1.495 (4)
Zn1—I1	2.6018 (4)	C7—H7A	0.9800
Zn1—I2	2.6506 (4)	C7—H7B	0.9800
O1—C11	1.423 (3)	C7—H7C	0.9800
O1—C12	1.426 (3)	C8—C9	1.521 (4)
N1—C1	1.332 (3)	C8—H8A	0.9900
N1—C5	1.349 (3)	C8—H8B	0.9900
N2—C6	1.277 (3)	C9—H9A	0.9900
N2—C8	1.459 (3)	C9—H9B	0.9900
N3—C9	1.479 (3)	C10—C11	1.521 (4)
N3—C13	1.490 (3)	C10—H10A	0.9900
N3—C10	1.491 (3)	C10—H10B	0.9900
C1—C2	1.394 (4)	C11—H11A	0.9900
C1—H1	0.9500	C11—H11B	0.9900
C2—C3	1.380 (4)	C12—C13	1.521 (4)
C2—H2	0.9500	C12—H12A	0.9900
C3—C4	1.394 (4)	C12—H12B	0.9900
C3—H3	0.9500	C13—H13A	0.9900
C4—C5	1.387 (4)	C13—H13B	0.9900
			
N2—Zn1—N1	74.71 (8)	C6—C7—H7B	109.5
N2—Zn1—N3	78.31 (8)	H7A—C7—H7B	109.5
N1—Zn1—N3	151.54 (8)	C6—C7—H7C	109.5
N2—Zn1—I1	137.01 (6)	H7A—C7—H7C	109.5
N1—Zn1—I1	95.53 (6)	H7B—C7—H7C	109.5
N3—Zn1—I1	98.03 (6)	N2—C8—C9	107.5 (2)
N2—Zn1—I2	109.52 (6)	N2—C8—H8A	110.2
N1—Zn1—I2	98.21 (6)	C9—C8—H8A	110.2
N3—Zn1—I2	99.18 (6)	N2—C8—H8B	110.2
I1—Zn1—I2	113.325 (12)	C9—C8—H8B	110.2
C11—O1—C12	110.8 (2)	H8A—C8—H8B	108.5
C1—N1—C5	118.9 (2)	N3—C9—C8	111.1 (2)
C1—N1—Zn1	126.70 (18)	N3—C9—H9A	109.4
C5—N1—Zn1	114.34 (17)	C8—C9—H9A	109.4
C6—N2—C8	122.6 (2)	N3—C9—H9B	109.4
C6—N2—Zn1	120.65 (18)	C8—C9—H9B	109.4
C8—N2—Zn1	116.61 (16)	H9A—C9—H9B	108.0
C9—N3—C13	111.1 (2)	N3—C10—C11	112.5 (2)
C9—N3—C10	112.5 (2)	N3—C10—H10A	109.1
C13—N3—C10	107.4 (2)	C11—C10—H10A	109.1
C9—N3—Zn1	100.98 (15)	N3—C10—H10B	109.1
C13—N3—Zn1	111.80 (16)	C11—C10—H10B	109.1
C10—N3—Zn1	113.10 (15)	H10A—C10—H10B	107.8
N1—C1—C2	122.6 (3)	O1—C11—C10	111.4 (2)
N1—C1—H1	118.7	O1—C11—H11A	109.3
C2—C1—H1	118.7	C10—C11—H11A	109.3
C3—C2—C1	118.3 (3)	O1—C11—H11B	109.3
C3—C2—H2	120.8	C10—C11—H11B	109.3
C1—C2—H2	120.8	H11A—C11—H11B	108.0
C2—C3—C4	119.6 (3)	O1—C12—C13	111.7 (2)
C2—C3—H3	120.2	O1—C12—H12A	109.3
C4—C3—H3	120.2	C13—C12—H12A	109.3
C5—C4—C3	118.4 (3)	O1—C12—H12B	109.3
C5—C4—H4	120.8	C13—C12—H12B	109.3
C3—C4—H4	120.8	H12A—C12—H12B	107.9
N1—C5—C4	122.1 (2)	N3—C13—C12	113.3 (2)
N1—C5—C6	114.8 (2)	N3—C13—H13A	108.9
C4—C5—C6	123.1 (2)	C12—C13—H13A	108.9
N2—C6—C5	115.4 (2)	N3—C13—H13B	108.9
N2—C6—C7	125.4 (2)	C12—C13—H13B	108.9
C5—C6—C7	119.2 (2)	H13A—C13—H13B	107.7
C6—C7—H7A	109.5		

### Hydrogen-bond geometry (Å, °)

**Table:**

*D*—H···*A*	*D*—H	H···*A*	*D*···*A*	*D*—H···*A*
C13—H13A···O1^i^	0.99	2.55	3.491 (3)	159

Symmetry codes: (i) −*x*+1, −*y*+2, −*z*+2.
